# Workplace Prosocial Activities and Daily Well-Being During COVID-19

**DOI:** 10.1093/geroni/igab046.2084

**Published:** 2021-12-17

**Authors:** Laura Carstensen, Kevin Chi

**Affiliations:** 1 Stanford University, Stanford University, Stanford, California, United States; 2 Stanford University, Stanford, California, United States

## Abstract

Workplace prosocial activities, such as providing unpaid assistance to colleagues, has been linked to better well-being. However, little is known about how these associations unfold in daily life. This study examines how prosocial activities at work are associated with daily well-being during the COVID-19 pandemic. A sample of 22 employees (aged 22-69 years) from a wealth management firm reported their daily activities and well-being on 10 consecutive workdays. On days when individuals provided help to someone they work with, they experienced higher positive affect, and greater enjoyment and interest at work, compared to days when they did not provide help. Individuals who provided more help reported greater meaning at work. Initial findings suggest that workplace prosocial activities have positive implications for daily well-being during the pandemic. Subsequent analyses will examine whether these findings replicate in a separate sample of working adults. Age differences in helping and meaning will be discussed.

